# Production of ROS by Gallic Acid Activates KDM2A to Reduce rRNA Transcription

**DOI:** 10.3390/cells9102266

**Published:** 2020-10-10

**Authors:** Yuji Tanaka, Hideru Obinata, Akimitsu Konishi, Noriyuki Yamagiwa, Makoto Tsuneoka

**Affiliations:** 1 Laboratory of Molecular and Cellular Biology, Faculty of Pharmacy, Takasaki University of Health and Welfare, Takasaki 370-0033, Japan; ytanaka@takasaki-u.ac.jp; 2 Education and Research Support Center, Gunma University Graduate School of Medicine, Maebashi 371-8511, Japan; obi@gunma-u.ac.jp; 3 Department of Biochemistry, Gunma University Graduate School of Medicine, Maebashi 371-8511, Japan; akimitsukonishi@gunma-u.ac.jp; 4 Laboratory of Molecular Design Chemistry, Faculty of Pharmacy, Takasaki University of Health and Welfare, Takasaki 370-0033, Japan; yamagiwa@takasaki-u.ac.jp

**Keywords:** rRNA transcription, breast cancer, KDM2A, gallic acid, ROS, histone demethylase

## Abstract

Metformin, which is suggested to have anti-cancer effects, activates KDM2A to reduce rRNA transcription and proliferation of cancer cells. Thus, the specific activation of KDM2A may be applicable to the treatment of cancers. In this study, we screened a food-additive compound library to identify compounds that control cell proliferation. We found that gallic acid activated KDM2A to reduce rRNA transcription and cell proliferation in breast cancer MCF-7 cells. Gallic acid accelerated ROS production and activated AMPK. When ROS production or AMPK activity was inhibited, gallic acid did not activate KDM2A. These results suggest that both ROS production and AMPK activation are required for activation of KDM2A by gallic acid. Gallic acid did not reduce the succinate level, which was required for KDM2A activation by metformin. Metformin did not elevate ROS production. These results suggest that the activation of KDM2A by gallic acid includes mechanisms distinct from those by metformin. Therefore, signals from multiple intracellular conditions converge in KDM2A to control rRNA transcription. Gallic acid did not induce KDM2A-dependent anti-proliferation activity in non-tumorigenic MCF10A cells. These results suggest that the mechanism of KDM2A activation by gallic acid may be applicable to the treatment of breast cancers.

## 1. Introduction

The ribosome is a unique machine for protein synthesis in organisms, and the number of ribosomes widely affects cellular activities [1,2]. Ribosome biogenesis consumes cellular materials supplied by nutrients and their metabolites, and the level of ribosome biogenesis reflects intracellular conditions. Therefore, cells should have mechanisms to control ribosome production by detecting intracellular conditions. The control of rRNA transcription is a major factor determining the amount of ribosome production [3], and thus, the levels of rRNA transcription affect multiple cellular activities including cell proliferation [4,5]. It was reported over a century ago that the number and sizes of nucleoli, the sites of ribosome biogenesis, are increased in cancer cells, and inhibition of rRNA transcription has been proposed as a therapeutic strategy against cancer cells [6].

The control of chromatin structure affects transcription and involves chemical modification of DNA and histones including the addition or removal of methyl groups [7,8]. Lysine-specific histone demethylase (KDM) consists of two main classes: a flavin adenine dinucleotide-dependent amine oxidase, and an Fe(II) and α-ketoglutarate-dependent hydroxylase that has the JmjC domain as an active center of the enzyme. The JmjC-type KDMs are one group of α-ketoglutarate-dependent oxygenases that catalyze a remarkably diverse range of oxidative reactions [9]. The JmjC-type KDMs remove methyl groups on lysine in the specific amino acid stretches of histones, and the selectivity of substrates is determined by the structure of the JmjC domain and also the dynamics of the amino acid sequence near the JmjC domain [10,11]. KDMs also distinguish the number of methyl groups attached to lysine [7]. Dysregulation of JmjC-type KDM family members is implicated in many diseases, including developmental disorders and cancers. Recently, increasing numbers of studies have suggested that epigenetic regulators control rRNA transcription [3,4]. We previously showed that a JmjC-type KDM, KDM2A, decreases the dimethylated lysine 36 of histone H3 (H3K36me2) but not trimethylated lysine 36 of H3 (H3K36me3) in the rRNA gene (rDNA) promoter, and represses rRNA transcription under starvation in breast cancer cells [12,13]. Further, we found that glucose depletion activates AMP-activated protein kinase (AMPK), which is required for KDM2A to reduce rRNA transcription and cell proliferation of breast cancer cells [12]. We also found that metformin, known as a treatment for type 2 diabetes, activates KDM2A to reduce rRNA transcription and cell proliferation [14]. A 4-h treatment with metformin not only activated AMPK, but also independently decreased the intracellular succinate level [14]. The activation of KDM2A by metformin required both AMPK activity and a decrease of the intracellular succinate level [14], suggesting that reduction of intracellular succinate, in addition to AMPK activation, is a mechanism by which KDM2A is activated to reduce rRNA transcription.

Gallic acid [3,4,5-trihydroxybenzoic acid], a natural botanic phenolic compound, is abundantly found in green tea, grapes, and red wine [15,16,17]. Gallic acid functions as an antioxidant to exert a wide range of pharmacological properties including anti-obesity, anti-inflammation, and anti-cancer activities [18]. On the other hand, reactive oxygen species (ROS) was also generated when gallic acid was added to the cell culture medium [19]. Gallic acid autoxidation produced significant levels of H_2_O_2_ and O_2_^• −^ (superoxide), and the number of apoptotic cells increased depending on the gallic acid concentration. Intracellular ROS levels that increased by gallic acid were reduced by *N*-acetyl-l-cysteine (NAC) and l-glutathione (GSH) [20]. Therefore, gallic acid can produce ROS in addition to its antioxidant activity.

Because KDM2A has the activity to reduce rRNA transcription and proliferation of breast cancer cells [12], we previously proposed that the reduction of rRNA transcription by KDM2A specifically in cancer cells may be applied to the treatment of breast cancers [21]. In this study, we screened a food-additive compound library to identify compounds that control cell proliferation. We found that gallic acid reduced rRNA transcription and cell proliferation in a KDM2A-dependent manner in the breast cancer cell line MCF-7 but not in non-tumorigenic MCF10A cells. Gallic acid accelerated ROS production and also activated AMPK. When the ROS production was reduced by GSH or NAC, gallic acid did not reduce rRNA transcription and cell proliferation. These results suggest that ROS is involved in the activation of KDM2A by gallic acid.

## 2. Materials and Methods

### 2.1. Cell Culture and Culture Medium

The human breast adenocarcinoma cell line MCF-7 was cultured in RPMI 1640 with l-Gln medium (Nacalai Tesque, Kyoto, Japan; #30264-85) supplemented with 10% fetal calf serum (FCS), 100 units/mL penicillin G (Nacalai Tesque; #26239-42), and 100 µg/mL streptomycin sulfate (Nakalai Tesque; #33204-92). The human non-tumorigenic epithelial cell line MCF10A was cultured in DMEM/Ham’s F-12 with l-Gln, sodium pyruvate, and HEPES, without phenol red (Nacalai Tesque; #05177-15) supplemented with 5% FCS, 0.5 μg/mL hydrocortisone, 0.1 μg/mL cholera toxin, 20 ng/mL EGF, 10 μg/mL insulin, 100 units/mL penicillin G (Nacalai Tesque; #26239-42), and 100 µg/mL streptomycin sulfate (Nakalai Tesque; #33204-92). Cells were maintained at 37 °C in a humidified atmosphere containing 5% CO_2_.

### 2.2. Measurement of Cell Proliferation

The cell numbers were analyzed using CyQUANT^®^ Direct Cell Proliferation Assay (ThermoFisher, Waltham, MA, USA; #C35011) according to the manufacturer’s instructions. In brief, cells plated in 96-well clear bottom microplates (Greiner, Kremsmenst, Austria; #655090) were treated with compounds as indicated. A cell-permeable DNA-binding dye in the kit was added, and cells were incubated for 1 h. The signals from cells were measured by an ARVO MX plate reader (PerkinElmer Inc., Waltham, MA, USA) using standard green filter sets from the bottom. The experiments were performed multiple times, the values were averaged, and the results were shown as a fold change to the values without the treatment with compounds.

### 2.3. siRNAs

Cells were transfected with siRNAs using Lipofectamine RNAiMAX (Thermofisher; #13778150) according to the manufacturer’s instructions with suitable amounts of siRNAs (150 pmol per 10 cm dish). The siRNA specific for KDM2A was 5′-GAACCCGAAGAAGAAAGGAUUCGUU-3′, which was previously described [12,13]. The control siRNA, Stealth™ RNAi Negative Control Medium GC Duplex (Life Technologies, Carlsbad, CA, USA), was purchased. Three days after transfection, these cells were used for experiments.

### 2.4. Total RNA Extraction and Quantitative Reverse Transcription-Polymerase Chain Reaction (qRT-PCR)

Total RNA was isolated from cells using a NucleoSpin RNA II kit (Takara Bio, Suozu, Japan; #U0955C) according to the manufacturer’s instructions. Single-strand cDNA was synthesized from total RNA (0.4 µg) by a Superscript III First-strand Synthesis system (Thermofisher; # 18080051) using random hexamers according to the manufacturer’s instructions. The products were diluted up to 100 µL with distilled water, and 2.5 µL of the resultant single-strand cDNA was used as the template for qRT-PCR using a KAPA SYBR FAST qPCR Master Mix Kit (KAPA Biosystems, Wilmington, MA, USA; #KR0389) with a CFX Connect Real-Time PCR Detection System (Bio-Rad Laboratories Inc., Hercules, CA, USA). The values measured by qRT-PCR were normalized by the amounts of β-actin mRNA and shown as a ratio of control conditions. To evaluate levels of rRNA transcription, the amounts of pre-rRNA were measured. The sets of PCR primers for amplification of the pre-rRNA were 5′-GCTGACACGCTGTCCTCTG-3′ and 5′-TCGGACGCGCGAGAGAAC-3′ (a sequence of 1–155 in pre-rRNA); for KDM2A, the primers were 5′-TCCCCACACACATTTTGACATC-3′ and 5′-GGGGTGGCTTGAGAGATCCT-3′; for β-actin, the primers were 5′-CGTCTTCCCCTCCATCGT-3′ and 5′-GAAGGTGTGGTGCCAGATTT-3′, as previously described [14].

### 2.5. ChIP Assay

The chromatin immunoprecipitation (ChIP) assay was performed as described previously [14] using Dynabeads^®^ protein G (ThermoFisher; #10003D). In brief, the cells were fixed with 1% formaldehyde for 10 min at 37 °C. After the addition of 0.125 M glycine and incubation for 5 min, cells were washed with PBS three times. Then, cells were lysed with ChIP lysis buffer (10 mM Tris-HCl (pH 8.0), 150 mM NaCl, 1%SDS, 1 mM EDTA and 0.2 mM PMSF) and sonicated. After centrifuging the cell lysate, the supernatant was diluted with the ChIP dilution buffer (20 mM Tris-HCl, 150 mM NaCl, 1 mM EDTA, 1% Triton X-100, and 0.2 mM PMSF). The diluted lysates were mixed with antibodies previously bound to Dynabeads protein G and incubated overnight at 4 °C. The next day, the beads were washed with Wash buffer 1 (20 mM Tris-HCl (pH 8.0), 500 mM NaCl, 2 mM EDTA, 1% Triton X-100, and 0.1% SDS) and Wash buffer 2 (10 mM Tris-HCl (pH 8.0), 250 mM LiCl, 1 mM EDTA, 0.5% Na-deoxycholate, and 0.5% NP-40). After two washes with TE buffer (10 mM Tris-HCl (pH 8.0) and 1 mM EDTA), the immunoprecipitated DNA was purified by a Chelex-100-based DNA isolation procedure [22], and the DNA fragments were quantified by qRT-PCR. The primers used for detecting the rDNA promoter (rDNA from +1 to +155 from the transcriptional start site; the same primers as used for pre-rRNA detection) were described previously [13]. To detect specific binding, the values obtained by specific antibodies were divided by input (% of input) and normalized by the values of the control antibody (normal rabbit IgG). When detecting histone modifications, the values for the specific binding were normalized by the values for H3 (% of specific bound/input normalized by H3). The experiments were repeated at least three times. The averages and standard deviations of the results are shown.

### 2.6. Antibodies

Anti-dimethylated histone H3 lys36 antibody (MAB Institute Inc., Sapporo, Japan; #MABI0332-100), anti-trimethylated histone H3 lys36 antibody (MAB Institute, Inc.; #MABI0333-100), anti-histone H3 antibody (Abcam, Cambridge, England; # ab1791), and the control antibody (normal rabbit IgG, Cell Signaling, Danvers, MA, USA; #2729S) for ChIP assays were purchased. The anti-KDM2A antibody was described previously [13]. Anti-phosphorylated AMPKα (Thr-172) and anti-AMPKα antibodies for immunoblotting were purchased (AMPK and ACC Antibody Sampler Kit, Cell Signaling; #9957). Anti-β-actin antibody was also purchased (Sigma AC-15, St. Louis, MO, USA; #A5441).

### 2.7. Immunoblotting

Cells were harvested after treatment in each experiment and extracted using an SDS-PAGE sampling buffer (4% SDS solution containing 100 mM Tris, pH 6.8, 50 mM DTT, and 20% glycerol) of suitable volume to adjust extracts to the same concentration. Cell extracts were separated by SDS-PAGE and transferred to a PVDF membrane (Millipore, Burlington, MA, USA; #IPVH00010). After treatment with antibodies, bands were detected using an Immobilon Western system (Millipore; #WBKLS0100) as described previously [13].

### 2.8. Agents

Compound C (IN Solution™ AMPK Inhibitor, compound C, Calbiochem, St. Louis, MO, USA; #171261) and SBI-0206965 (Sigma; #SML1540) [14] were purchased. The food-additive compound library was purchased (Sigma #S990043-FDS1). Gallic acid (Nacalai Tesque, Kyoto, Japan; #16520-42) was purchased and dissolved in ethanol at 0.5 M for the stock solution. The N-acetylcysteine (NAC, Nacalai Tesque; #11568-92), glutathione (GSH, Nacalai Tesque; #08786-61), hydrogen peroxide (H_2_O_2,_ Wako Chemical, Richmond, VA, USA; #081-04215), and dimethyl-succinate (DMS) (TCI, Palo Alto, CA, USA; #S0104; succinic acid dimethyl ester) were purchased.

### 2.9. DCFDA Assay for Intracellular ROS Detection

DCFDA assays were performed using a DCFDA/H2DCFDA-Cellular ROS Assay Kit (Abcam; #ab113851) according to the manufacturer’s instructions. In brief, 20,000 cells plated in 96-well clear bottom microplates (Greiner #655090) were washed with the buffer in the kit and treated with 20 μM DCFDA probe for 45 min at 37 °C in a humidified atmosphere containing 5% CO_2_. DCFDA was converted to DCF inside cells. After washing cells with PBS once, cells were placed in the culture medium in the presence of various compounds, and DCF signals were measured at various times. The DCF signals were measured using an ARVO MX plate reader (PerkinElmer Inc.) using standard green filter sets (Excitation/Emission: 485/535 nm) from the bottom. The values of blanks, which contained no cells and compounds, were subtracted from DCF signal values.

### 2.10. Detection of Intracellular Succinate, α-KG, and Citrate Levels

The intracellular levels of succinate and α-KG in methanol extracted from cells were measured by liquid chromatography-tandem mass spectrometry (LC-MS/MS) as previously described [14].

### 2.11. Statistics

Sample sizes and error bars are indicated in each figure. The *p*-values were calculated with one-way ANOVA (Tukey HSD) by using statistical software EZR based on R and R Commander [23].

## 3. Results

### 3.1. Gallic Acid Induced KDM2A Dependent-Reduction of rRNA Transcription and Cell Proliferation in MCF-7 Cells

**Figure 1:**
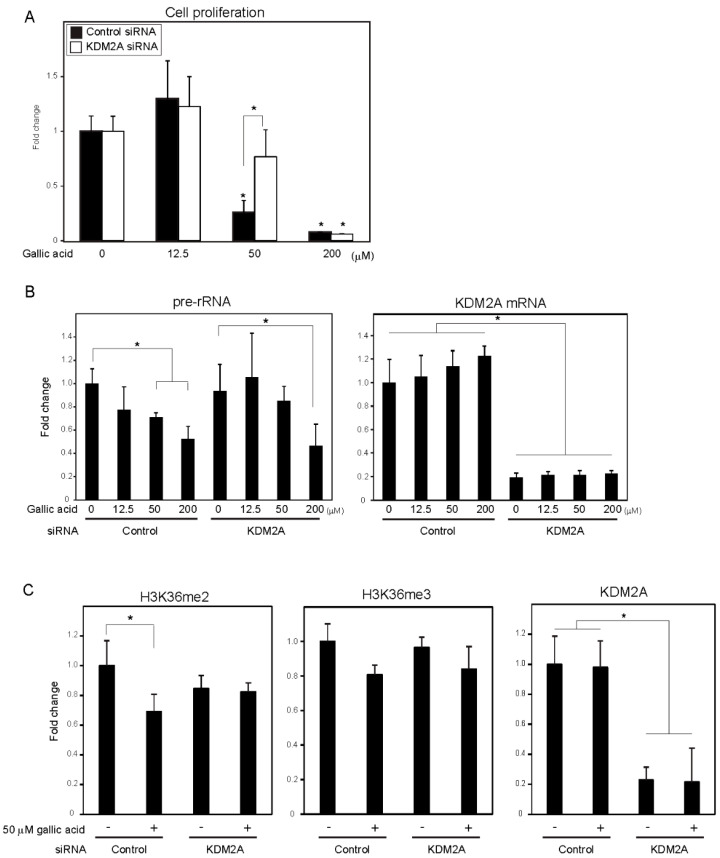
Gallic acid induced KDM2A-dependent reductions of cell proliferation, rRNA transcription, and H3K36me2 in rDNA promoter in MCF-7 cells. (**A**) Gallic acid decreased proliferation of MCF-7 cells through KDM2A. MCF-7 cells transfected with control siRNA or siRNA for KDM2A were cultured with gallic acid as indicated concentrations for 2 days. Cell numbers were measured by CyQUANT^®^ Direct Cell Proliferation Assay detecting DNA content. (**B**) The treatment with 50 μM gallic acid reduces rRNA transcription through KDM2A. MCF-7 cells transfected with control siRNA or siRNA for KDM2A were treated with gallic acid at indicated concentrations for 4 h. Total RNAs were isolated and analyzed by quantitative real-time PCR (qRT-PCR) to detect rRNA transcription (pre-rRNA) (left panel) and KDM2A mRNA (right panel). The ratios of the values for cells treated with various conditions to those for cells treated with control siRNA without gallic acid are shown. (**C**) KDM2A-dependent reduction of H3K36me2 marks in rDNA promoter by treatment with 50 μM gallic acid. MCF-7 cells transfected with control siRNA or siRNA for KDM2A were treated with 50 μM gallic acid for 4 h. The levels of H3K36me2, H3K36me3, and KDM2A in the rDNA promoter were analyzed by ChIP assay. The results are expressed as fold changes of the values under various conditions to those in cells cultured with control siRNA without gallic acid treatment. All experiments were performed more than three times, and the mean values with standard deviations are indicated. * *p* < 0.05.

A food-additive compounds library (Sigma; #S990043-FDS1) was used to screen compounds that control cell proliferation of breast cancer cell line MCF-7 cells. Cells were treated with 133 μM of each compound for 2 days, and cell numbers were detected with CyQUANT Direct Cell Proliferation Assay (Figure S1A) by measuring the amounts of DNA. Nine out of 77 compounds decreased cell numbers to less than 80%, and 15 compounds tended to increase cell numbers. Among the compounds, gallic acid reduced cell numbers in a KDM2A-dependent manner (Figure S1B). Therefore, we focused our study on the mechanism of gallic acid to decrease cell proliferation of MCF-7 cells. When MCF-7 cells were treated with various concentrations of gallic acid for 2 days, treatment with 50 or 200 μM gallic acid decreased the cell number, but 12.5 μM gallic acid did not (Figure 1A). The KDM2A knockdown significantly alleviated the decrease of cell number by 50 μM gallic acid, but not that by 200 μM gallic acid (Figure 1A). These results suggested that the treatment with 50 μM gallic acid decreased cell proliferation through KDM2A.

To investigate whether the decrease of cell numbers by gallic acid was associated with the decrease of rRNA transcription, the levels of rRNA transcription were measured at 4 h after gallic acid treatment. The treatment of cells with gallic acid decreased rRNA transcription in a dose-dependent manner (Figure 1B), and the KDM2A knockdown alleviated the decrease of rRNA transcription in cells treated with 50 μM gallic acid (Figure 1B). In the case of 200 μM gallic acid, the levels of rRNA transcription were reduced even when KDM2A was knocked down (Figure 1B). Treatment with 50 μM gallic acid decreased the level of H3K36me2, a direct substrate of KDM2A, in the rDNA promoter, depending on KDM2A (Figure 1C), but did not significantly affect the levels of neither KDM2A nor H3K36me3 in the rDNA promoter (Figure 1C). The demethylation of JmjC-type enzymes proceeded by a side reaction that produced succinate from α-ketoglutarate (α-KG) [24], and it was shown that succinate can inhibit the demethylase activity of KDM2A [12,13,14]. The addition of a cell-permeable succinate, dimethyl succinate (DMS), to the medium inhibited the reductions of H3K36me2 in the rDNA promoter and rRNA transcription induced by 50 μM gallic acid (Figure S2). These results suggest that 50 μM gallic acid activated the demethylase activity of KDM2A to reduce rRNA transcription and cell proliferation.

### 3.2. Gallic Acid Elevated ROS Production and AMPK Activation, both of which are Required for KDM2A to Regulate H3K36me2 Levels in the rDNA Promoter and rRNA Transcription

**Figure 2:**
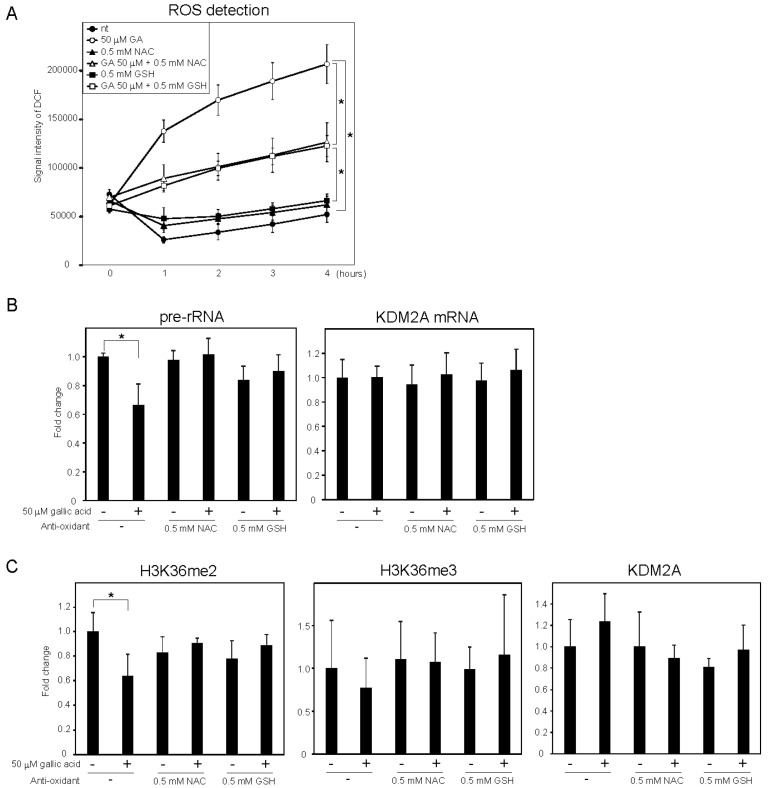
ROS production by gallic acid was required for the repression of rRNA transcription mediated by KDM2A in MCF-7 cells. (**A**) Gallic acid increases ROS production in MCF-7 cells. MCF-7 cells cultured with cell-permeable ROS probe DCFDA were cultured with or without 50 μM gallic acid (GA) in the presence or absence of 0.5 mM *N*-acetyl-l-cysteine (NAC) or l-glutathione (GSH). At the indicated times, the increases in the signal intensities of DCF by ROS in cells were measured. (**B**) Effects of anti-oxidants, NAC and GSH, on the reduction of rRNA transcription by gallic acid. MCF-7 cells treated with or without 50 μM gallic acid in the presence or absence of 0.5 mM NAC or 0.5 mM GSH for 4 h. Total RNAs were isolated and analyzed by qRT-PCR to detect pre-rRNA (left panel) and KDM2A mRNA (right panel). The ratios of the values for cells treated with various conditions to those for cells treated without gallic acid and anti-oxidants are shown. (**C**) Effects of anti-oxidants, NAC and GSH, on the reduction of H3K36me2 marks in the rDNA promoter by gallic acid. MCF-7 cells were cultured for 4 h with or without 50 μM gallic acid in the presence or absence of NAC or GSH. The levels of H3K36me2, H3K36me3, and KDM2A in the rDNA promoter were analyzed by ChIP assays. The results are expressed as fold changes to the values in various conditions to those without gallic acid and anti-oxidants. All experiments were performed more than three times, and the mean values with standard deviations are indicated. * *p* < 0.05.

It was reported that gallic acid showed anti-cancer activity in some cancer cells that probably involved the production of ROS [25,26]. We measured the levels of intracellular ROS using 2′,7′-dichlorofluorescein (DCF) diacetate, a cell-permeable probe. It was found that treatment with 50 μM gallic acid increased the DCF signal (Figure 2A). Antioxidants, such as N-acetylcysteine (NAC) and glutathione (GSH), reduced the DCF signal increased by 50 μM gallic acid (Figure 2A). These results show that gallic acid treatment increased the level of intracellular ROS in MCF-7 cells. The NAC and GSH treatments impaired the reduction of rRNA transcription (Figure 2B) and H3K36me2 marks in the rDNA promoter (Figure 2C) induced by 50 μM gallic acid. The levels of H3K36me3 and KDM2A in the rDNA promoter were not significantly changed under these conditions (Figure 2C). The results indicate that the increase of ROS by gallic acid is required for the induction of KDM2A activity to reduce rRNA transcription.

Next, whether the oxidative stress alone repressed rRNA transcription through KDM2A was tested. When cells were treated with various concentrations of H_2_O_2_, rRNA transcription was reduced and the KDM2A knockdown slightly alleviated the reduction of rRNA transcription at 12.5 μM H_2_O_2_ (Figure S3A). However, the level of H3K36me2 in the rDNA promoter was not reduced by 12.5 μM H_2_O_2_ (Figure S3B). Therefore, H_2_O_2_ alone did not activate the KDM2A demethylase activity in the rDNA promoter.

**Figure 3:**
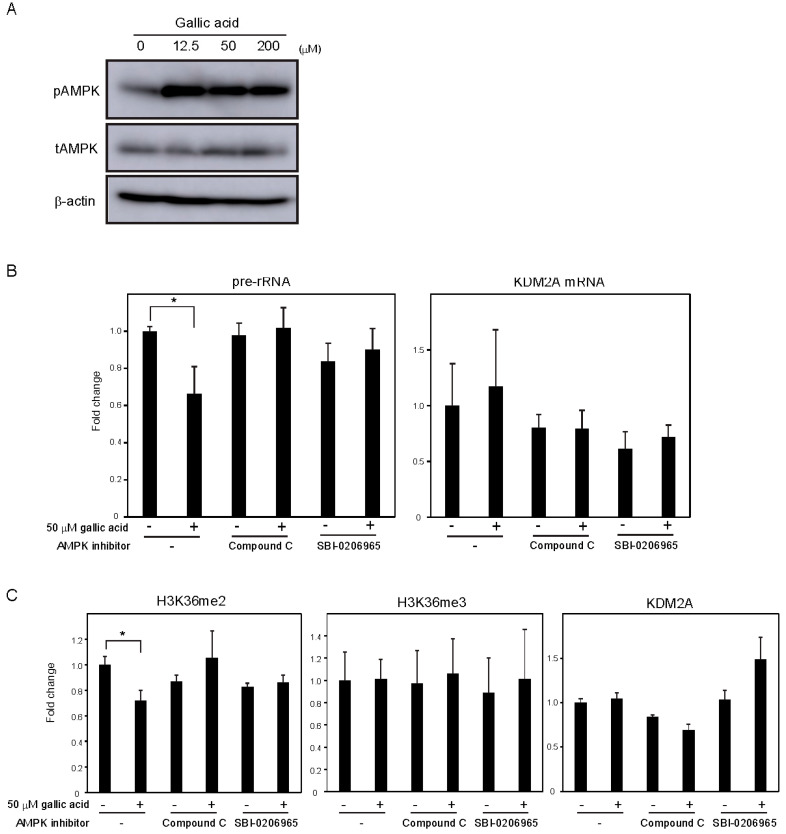
AMPK activation is required for the KDM2A-mediated repression of rRNA transcription by gallic acid in MCF-7 cells. (**A**) Activation of AMPK by gallic acid in MCF-7 cells. MCF-7 cells were treated with gallic acid as indicated concentrations for 4 h. Cells were lysed and analyzed for the levels of phosphorylated-AMPKα (Thr172) (pAMPK), total AMPKα (tAMPK), and β-actin by immunoblotting. (**B**) Requirement of AMPK activity for the reduction of rRNA transcription by gallic acid. MCF-7 cells treated with or without 50 μM gallic acid in the presence or absence of AMPK inhibitor, 10 μM compound C or 5 μM SBI-0206965, for 4 h. Total RNAs were isolated and analyzed by qRT-PCR to detect pre-rRNA (left panel) and KDM2A mRNA (right panel). The ratios of the values for cells treated with various conditions to those for cells treated without gallic acid and AMPK inhibitors are shown. The experiments were performed three times (*n* = 3), and the mean values with standard deviations are indicated. * *p* < 0.05. (**C**) Requirement of AMPK activity for the reduction of H3K36me2 mark in the rDNA promoter by gallic acid. MCF-7 cells treated with or without 50 μM gallic acid in the presence of 10 μM compound C or 5 μM SBI-0206965 for 4 h. The levels of H3K36me2, H3K36me3, and KDM2A in the rDNA promoter were analyzed by ChIP assay. The results are expressed as fold changes to the values in various conditions to those without gallic acid and AMPK inhibitors. The experiments were performed three times (*n* = 3), and the mean values with standard deviations are indicated. * *p* < 0.05.

Previously, we showed that AMPK activity was required for KDM2A to reduce the levels of H3K36me2 in the rDNA promoter and rRNA transcription under glucose starvation [12] or by metformin [14]. Treatment with gallic acid was reported to activate AMPK in the liver cancer cell line HepG2 cells [27]. When MCF-7 cells were treated with 0, 12.5, 50, or 200 μM gallic acid for 4 h, the level of Thr172 phosphorylated AMPKα, the activation mark of AMPK, was increased (Figure 3A, pAMPK). The amounts of total AMPKα (tAMPK) and β-actin were not changed under these conditions (Figure 3A, tAMPK), showing that the treatment with gallic acid activated AMPK. Treatments with compound C or SBI-0206965, known as inhibitors of AMPK, impaired the 50 μM gallic acid-dependent reductions of rRNA transcription (Figure 3B) and H3K36me2 in the rDNA promoter (Figure 3C). These results suggest that the activation of AMPK by 50 μM gallic acid is required for the reduction of rRNA transcription through KDM2A. The treatments with NAC and GSH did not impair AMPK activation by 50 μM gallic acid (Figure S4), showing that gallic acid causes the AMPK activation without elevating ROS production. Together, these results suggest that gallic acid elevates AMPK activation and ROS production, both of which are required for KDM2A to regulate rRNA transcription through its histone demethylase activity in the rDNA promoter.

### 3.3. Activation of KDM2A by Gallic Acid Included Different Mechanisms from those by Metformin

Demethylation of JmjC-type enzymes proceeds with a side reaction that produces succinate from α-ketglutarate (α-KG) [19]. Therefore, reduction of succinate and/or elevation of α-KG positively affected KDM2A activity [14]. Previously, we reported that treatment with metformin for 4 h decreased the amounts of intracellular succinate and that reduction of succinate levels is required to activate KDM2A by metformin in addition to AMPK activation [14]. When the levels of succinate were measured after treatment with gallic acid for 4 h, it was found that gallic acid hardly decreased the succinate level (Figure S5A), while treatment with 2.5 mM metformin for 4 h did decrease it (Figure S5A). Gallic acid also did not affect the level of α-KG. These results suggest that gallic acid affects KDM2A activity independently of the levels of intracellular succinate and α-KG. On the other hand, ROS production was not elevated by treatment of the cells with 2.5 mM or 10 mM metformin or an AMPK activator (0.5 mM AICAR) for 4 h, all of which are capable of activating KDM2A [12,14] (Figure S5B). These results suggest that the activation of KDM2A by gallic acid involves different mechanisms from those by metformin, although both pathways require the activation of AMPK.

### 3.4. Gallic Acid Did Not Induce KDM2A-Dependent Anti-Cell Proliferation Activity in Non-Tumorigenic MCF10A Cells

**Figure 4:**
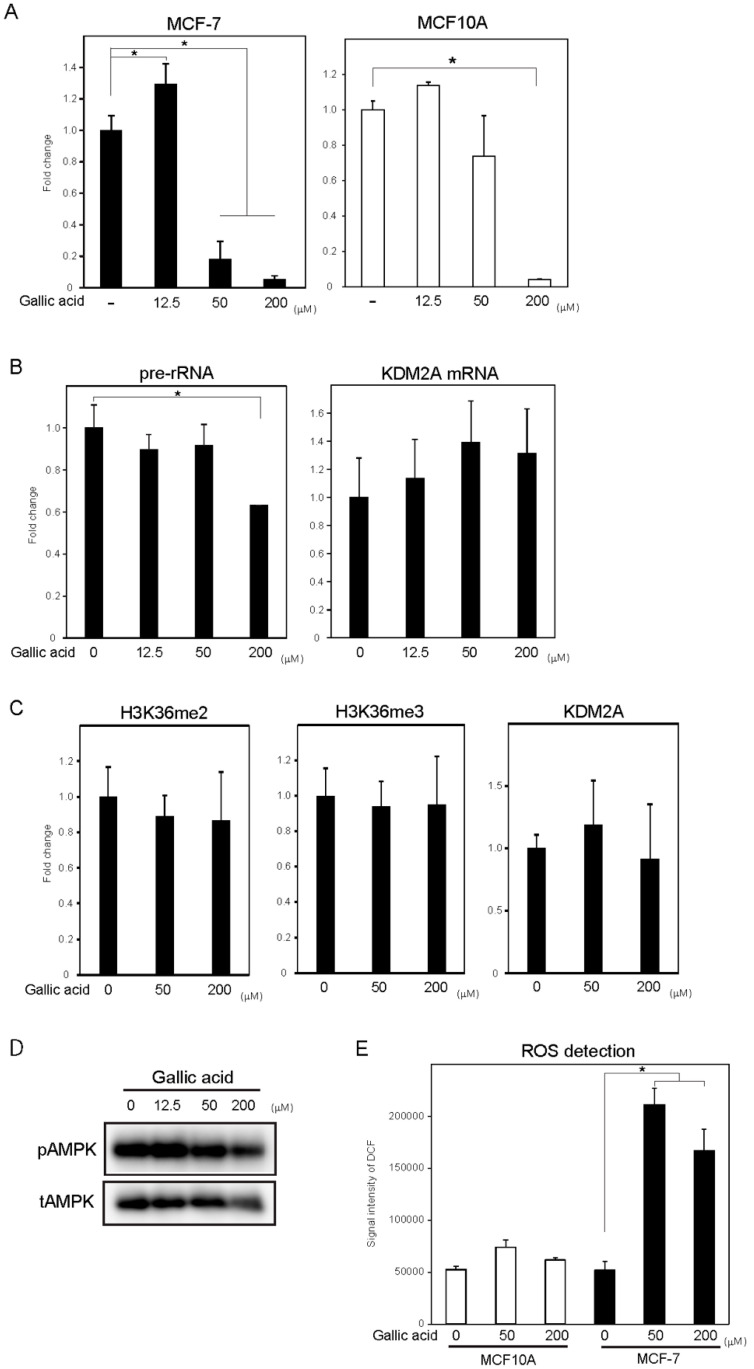
Gallic acid does not induce KDM2A activity to reduce cell proliferation in non-tumorigenic MCF10A cells. (**A**) The effects of gallic acid on cell proliferation of MCF10A cells. MCF-7 cells and MCF10A cells were treated with gallic acid as indicated concentrations. After culture for 2 days, cell numbers were analyzed by the same method as Figure 1A. The experiments were performed four times (*n* = 4), and the mean values with standard deviations are indicated. * *p* < 0.05. (**B**) The effects of gallic acid on rRNA transcription in MCF10A cells. The MCF10A cells were treated with gallic acid as indicated concentrations for 4 h. Total RNAs were isolated and analyzed by qRT-PCR to detect pre-rRNA (left panel) and KDM2A mRNA (right panel). The ratios of the values for cells treated with various concentrations of gallic acid to those for cells treated without gallic acid are shown. The experiments were performed three times (*n* = 3), and the mean values with standard deviations are indicated. * *p* < 0.05. (**C**) The effects of gallic acid on H3K36me2 mark in the rDNA promoter of MCF10A cells. MCF10A cells were treated with gallic acid at indicated concentrations for 4 h. The levels of H3K36me2, H3K36me3, and KDM2A in the rDNA promoter were analyzed by ChIP assay. The results are expressed as fold changes to the values with gallic acid to those without gallic acid. The experiments were performed three times (*n* = 3), and the mean values with standard deviations are indicated. (**D**) The effects of gallic acid on AMPK activation in MCF10A cells. MCF10A cells were treated with gallic acid as indicated concentrations for 4 h. Cells were lysed and the levels of phosphorylated-AMPKα (Thr172) (pAMPK) and total AMPKα (tAMPK) were analyzed by immunoblotting. (**E**) The effect of gallic acid on ROS production in MCF10A cells. MCF-7 cells and MCF10A cells were treated with or without gallic acid for 4 h. Cells were analyzed for ROS production by DCFDA probe as in Figure 2A. The experiments were performed four times (*n* = 4), and the mean values with standard deviations are indicated. * *p* < 0.05.

To test whether KDM2A in non-tumorigenic cells was activated by gallic acid, breast epithelial MCF10A cells were treated with gallic acid. Treatment with 50 μM gallic acid, which reduced cell proliferation of tumorigenic MCF-7 cells, did not reduce proliferation of MCF10A cells (Figure 4A, right panel). Although treatment of MCF10A cells with 200 μM gallic acid reduced the cell number, this reduction was not alleviated by a KDM2A knockdown (Figure S6). Treatment with 50 μM gallic acid also did not reduce rRNA transcription in MCF10A cells (Figure 4B). The levels of H3K36me2 as well as H3K36me3 and KDM2A in the rDNA promoter were not significantly changed by the treatment with 50 μM and 200 μM gallic acid (Figure 4C). These results suggested that gallic acid did not activate KDM2A in MCF10A cells to affect cell proliferation.

Next, the effects of gallic acid on AMPK activity and ROS production were investigated in MCF10A cells. The treatment with gallic acid hardly increased phosphorylated AMPK in MCF10A cells (Figure 4D). The elevation of ROS production by treatment with 50 or 200 μM gallic acid in MCF10A cells was far lower than those in MCF-7 cells (Figure 4E). These results suggest that tumorigenic MCF-7 cells are sensitive to gallic acid, to affect both ROS production and AMPK activation more than in non-tumorigenic MCF10A cells.

## 4. Discussion

### 4.1. Gallic Acid Induced ROS Production and AMPK Activation, both of which Contribute to Activation of KDM2A to Reduce rRNA Transcription

We previously proposed that the reduction of rRNA transcription by KDM2A may be applied to the treatment of breast cancers [21]. In this study, a food-additive compound library was screened to identify the compounds that control cell proliferation of breast cancer MCF-7 cells. We found several compounds that reduced cell proliferation of MCF7 cells. Among them, gallic acid reduced cell numbers in a KDM2A-dependent manner (Figure 1). Treatment with 50 μM gallic acid reduced rRNA transcription and cell proliferation in a KDM2A-dependent manner (Figure 1) and reduced the level of the KDM2A-substrate H3K36me2 in the rDNA promoter; further, a KDM2A knockdown inhibited the reduction. Cell-permeable succinate DMS, which inhibits the demethylase activity of KDM2A, inhibited the reductions induced by gallic acid (Figure S2). These results indicate that gallic acid activates demethylase activity of KDM2A in the rDNA promoter. Interestingly, the KDM2A-dependent reductions of rRNA transcription and cell proliferation induced by gallic acid were not observed in non-tumorigenic MCF10A cells (Figure 4), suggesting that this compound may function as a KDM2A activator specifically in cancerous cells.

It was reported that gallic acid produces ROS [25,26], and ROS widely affects cellular activities. We found that the gallic acid treatment increased intracellular ROS (Figure 2). The NAC or GSH treatment, which reduced the intracellular level of ROS, impaired the reductions of rRNA transcription and the H3K36me2 level in rDNA promoter induced by 50 μM gallic acid (Figure 2). These results suggest that the ROS increase by gallic acid is required for induction of KDM2A activity to reduce rRNA transcription by gallic acid. However, oxidative stress by H_2_O_2_ alone did not activate the KDM2A demethylase activity in the rDNA promoter (Figure S3). We found that gallic acid activated AMPK, and AMPK activity was required for the activation of KDM2A by gallic acid (Figure 3). There results show that gallic acid elevates ROS production and AMPK activation, both of which are required for the activation of KDM2A to reduce rRNA transcription.

ROS production was not elevated by metformin or an AMPK activator (Figure S5), suggesting that gallic acid is a unique compound that enhances ROS production among the KDM2A activators. The treatment with NAC or GSH did not impair AMPK activation by 50 μM gallic acid (Figure S4), suggesting that ROS production is not required for activation of AMPK. These results suggest that ROS production by gallic acid includes a distinct mechanism from that of AMPK activation.

### 4.2. Multiple Intracellular Signals Converge to Activate KDM2A to Control rRNA Transcription

The levels of rRNA transcription are regulated by many environmental and intracellular conditions. We have shown that 2DG, metformin, and gallic acid activate KDM2A to reduce rRNA transcription [12,14]. In these conditions, the activation of AMPK plays a critical role [12,14]. The reduction of intracellular succinate by metformin is an important factor in the activation of KDM2A [14]. We also reported that reduction of α-KG by culturing cells in a glutamine-free medium inhibits the activation of KDM2A by metformin [14]. While gallic acid neither reduced the succinate level nor elevated the α-KG level (Figure S5), gallic acid elevated ROS production, which was required for activation of KDM2A. In contrast, metformin did not elevate ROS production. Together, these results suggest that gallic acid activates KDM2A partly by mechanisms distinct from those of metformin. These results suggest that signals from multiple intracellular conditions, including the energy status detected by AMPK, metabolites in the TCA cycle, and redox status, converge to control the KDM2A activity that regulates rRNA transcription, and thus, suggest that KDM2A may be a central factor to finely tune rRNA transcription.

Recent studies suggest that KDM2A is involved in DNA damage response [28,29]. It has been reported that ataxia telangiectasia mutated specifically phosphorylates KDM2A at threonine 632 following DNA damage, and this phosphorylation abrogates the chromatin binding activity of KDM2A. Consequently, H3K36me2 near DNA damage sites is increased, and enriched H3K36me2 serves as a platform to recruit DNA repair complex to DNA damage sites [28]. Their immunoblotting assays of the cell extracts from HEK293T cells showed that H3K36me2 significantly increased on DNA damage response [28]. When extracts of MCF-7 and MCF10A cells were examined, H3K36me2 did not increase by gallic acid treatment (Figure S7A). DNA damage results in rapid phosphorylation of H2A.X at Ser139 (γH2A.X) [30]. The level of γH2A.X was not elevated by gallic acid treatment (Figure S7B). A “guardian of the genome”, p53, arrests cell cycle in response to DNA damage, in part by transcriptionally inducing the cyclin-dependent kinase inhibitor p21 [31]. MCF-7 cells express functional p53 [32]. The downstream target of p53, p21, was not increased by gallic acid treatment in MCF-7 cells (Figure S7B). MCF10A cells express functional p53 [33], but p21 was hardly detected in the conditions here (Figure S7B). Together, these results suggest that reduction of cell proliferation by gallic acid is not due to DNA damage response.

How ROS production by gallic acid activates KDM2A is an open question. One possibility is that the reaction proceeded by the JmjC domain is directly stimulated by ROS. Studies of the detailed reaction mechanism of KDMs show dioxygen binding in the active site of a JmjC histone demethylase [34,35]. This dioxygen binding plays a critical role and shows a strong dependence on the protein environment [10]. According to the model presented [10,34,35], ROS could directly affect this process. Another possibility is modifications of proteins that affect the activity of KDMs. Protein oxidative modifications are caused by reactions between amino acid residues and ROS. The oxidatively modified protein adducts could be produced by carbonylation, s-sulfenylation, s-nitrosylation, s-glutathionylation, and disulfide formation [36]. These chemical modifications in and around the JmjC domain may accelerate the KDM2A enzyme reactions, including the dioxygen binding in the active site of the JmjC domain.

### 4.3. Inhibition of rRNA Transcription by KDM2A activated by Gallic Acid may be Applicable to Cancer Treatment

There are several reports that show a positive effect of KDM2A on cell proliferation. The KDM2A-KO mice exhibited embryonic lethality at E10.5–12.5, accompanied with severe growth defects leading to reduced body size. Knockout of KDM2A decreased cell proliferation and increased apoptosis. The lack of KDM2A resulted in downregulation of the Polycomb group protein (PcG) Ezh2, PcG-mediated H2A ubiquitination, and upregulation of p21 [37]. The elevated expression of KDM2A was reported and promoted lung tumorigenesis by epigenetically enhancing ERK1/2 signaling [38]. FH-catalyzed fumarate in promoter regions inhibited KDM2A demethylase activity, and thus, maintained the H3K36me2 profile and facilitated gene expression for cell growth arrest [39]. Acceleration of cell growth by KDM2A was also observed in breast cancer cells [40]. These results suggest that KDM2A positively regulates cell proliferation under non-stressful conditions through controlling the expression of several genes. The experiments in this study were performed to detect KDM2A activity under stressful conditions, where AMPK was activated [12,13,14,21,41]. Under these conditions, KDM2A shows a negative effect on cell proliferation through reduction of rRNA transcription. These results suggest that KDM2A exerts both positive and negative effects on cell proliferation, depending on environmental and intracellular conditions. The positive effect of KDM2A on cell proliferation under non-stressful conditions could make cells maintain the expression of KDM2A in cancerous cells. This speculation is consistent to the finding of the continuous expression of KDM2A during carcinogenesis in breast carcinomas [12]. The presence of KDM2A in cancerous cells would give an advantage to the treatment of cancers though the activation of KDM2A to reduce rRNA transcription.

How demethylation of H3K36me2 in the rDNA promoter induces the reduction of rRNA transcription is not clear. Recently, some reports suggest that certain KDMs have multiple substrates, including non-histone proteins and potentially methylated arginyl residues [42,43]. KDM4s have demethylase activity on non-core histone, histone 1 isotype 4 methylated, as well as the well-established substrates H3K9me3 and H3K36me3 [44]. Therefore, there might be another substrate for KDM2A besides H3K36me2. In any case, our results demonstrate that gallic acid activates the demethylase activity of KDM2A to reduce cell proliferation.

Our results suggest that gallic acid activates KDM2A specifically in tumorigenic cells but not in non-tumorigenic cells. Therefore, gallic acid is a promising candidate for the treatment of breast cancers. Although treatment with 50 μM gallic acid reduced rRNA transcription and cell proliferation in a KDM2A-dependent manner in tumorigenic MCF-7 cells, higher concentrations of gallic acid did so in both tumorigenic and non-tumorigenic cells in a KDM2A-independent manner. Therefore, the concentration range of gallic acid to effectively activate KDM2A is limited, which may be an obstacle to applying this tumorigenic cell-specific activation of KDM2A for cancer treatment. One way to circumvent this problem is further screening of compounds that activate KDM2A. We have already found several compounds besides gallic acid that reduce MCF-7 cell proliferation (Figure S1). Studies of these compounds may identify a compound that could be easily applied for the treatment of breast cancers. Further investigation of the mechanisms by which KDM2A is activated by gallic acid could provide ways to improve the specific activation of KDM2A in tumorigenic cells.

## Supplementary Materials

The following are available online at https://www.mdpi.com/2073-4409/9/10/2266/s1, Figure S1: Screening of the chemical library to identify compounds that control proliferation of MCF-7 cells, Figure S2: DMS inhibits the activity of KDM2A activated by 50 μM gallic acid in MCF-7 cells, Figure S3: H_2_O_2_ does not activate the demethylase activity of KDM2A, Figure S4: AMPK activation occurs by 50 μM gallic acid in the presence of NAC and GSH, Figure S5: Intracellular succinate levels and ROS production in cells treated with gallic acid or metformin, Figure S6: Gallic acid does not reduce rRNA transcription through KDM2A in MCF10A cells, Figure S7: The effects of gallic acid on the levels of histone H3K36me2, p21 and γH2A.X.
