# Supplementary material for: Production of ROS by Gallic Acid Activates KDM2A to Reduce rRNA Transcription
Source: Cells. 2020 Oct 10;9(10):2266. doi: 10.3390/cells9102266 (PMC7601038; doi:10.3390/cells9102266)
Supplement: Supplementary file 1 [file cells-09-02266-s001.pdf]

cells MDPI

Supplementary figures: The following are available online at www.mdpi.com/2073-4409/9/10/2266/s1,

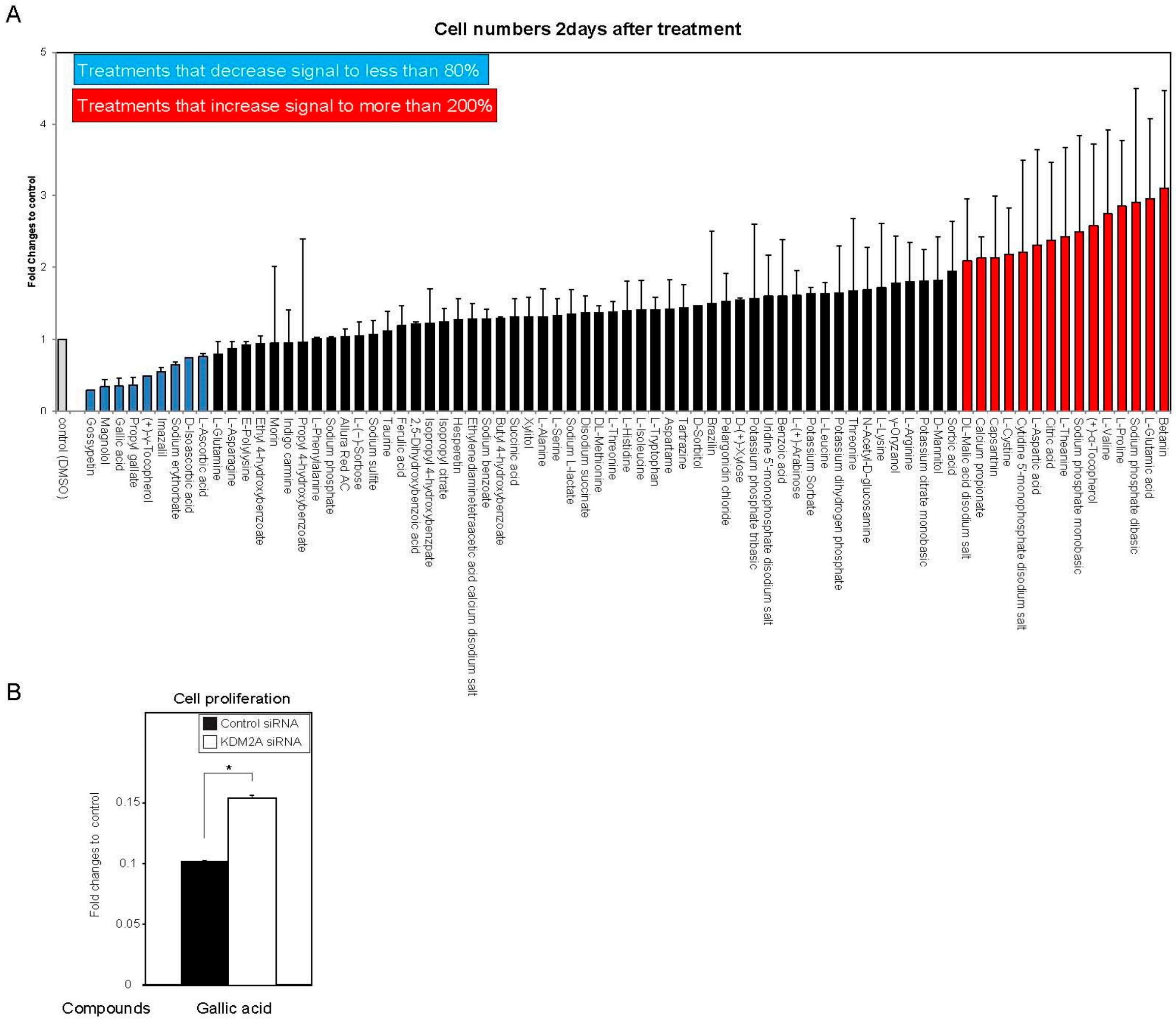

Figure S1 Tanaka et al.

Figure S1. Screening of the chemical library to identify compounds that control proliferation of MCF-7 cells. A, MCF-7 cells were plated in 96-well plates at one thousand cells per well. The next day, compounds in the food-additive compounds library (Sigma #S990043-FDS1) were added to wells at a concentration of 133 μM. After culturing for 2 days, cell numbers were evaluated by a CyQUANT Direct Cell Proliferation Assay to measure the DNA amounts. The results were shown as comparison of fold changes of the values with compounds to that with solvent (DMSO). N=2. B, MCF-7 cells transfected with control siRNA or siRNA for KDM2A were treated with 133 μM gallic acid. After culturing for 2 days, cell were counted by a CyQUANT® Direct Cell Proliferation Assay. The results are shown as fold changes of the values with compounds to that with solvent (DMSO). The experiments were performed three times (n=3), and the mean values with standard deviations are indicated. *; P<0.05.

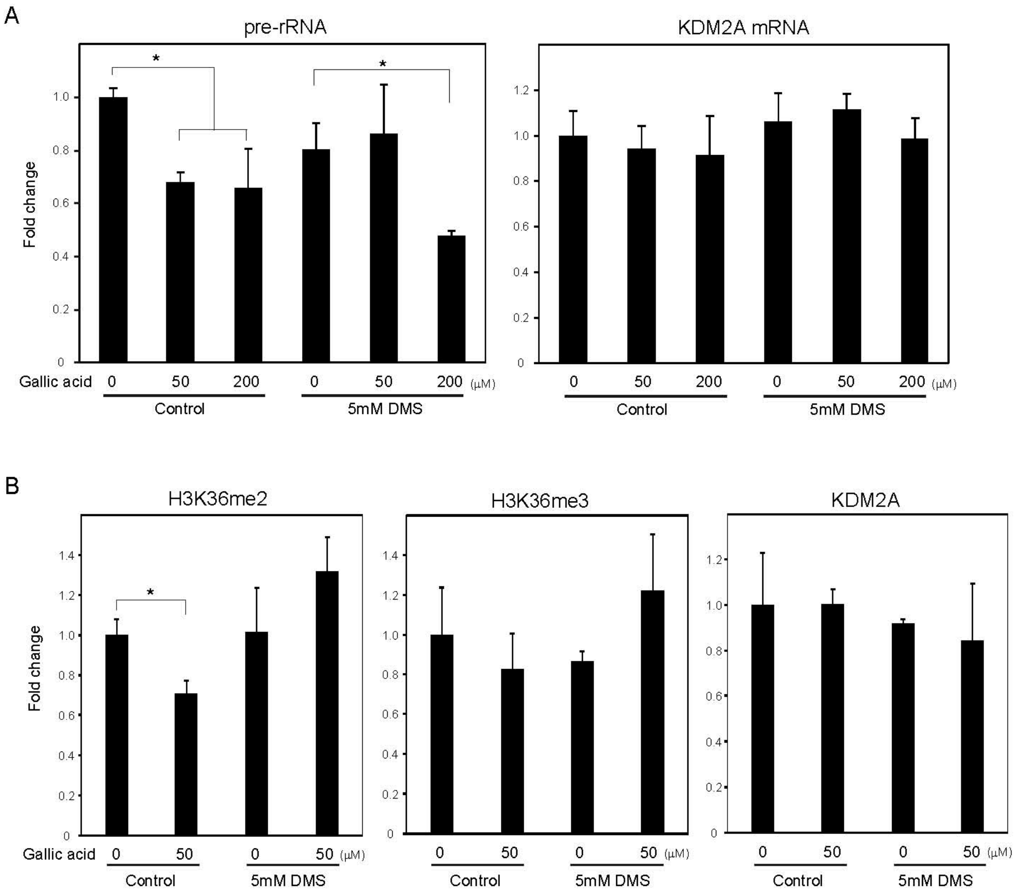

Figure S2 Tanaka et al.

Figure S2. DMS inhibits the activity of KDM2A activated by 50 μM gallic acid in MCF-7 cells. A, DMS treatment inhibits the repression of rRNA transcription by gallic acid. MCF-7 cells were treated with or without gallic acid in the presence or absence of 5 mM DMS. After 4 hours, total RNAs were isolated and analyzed by quantitative real-time PCR (qRT-PCR) to detect rRNA transcription (pre-rRNA) (left panel) and KDM2A mRNA (right panel). The ratios of the values for cells treated with gallic acid and/or DMS to those for cells treated without gallic acid and DMS are shown. B, DMS treatment inhibited the decrease of H3K36me2 mark in rDNA promoter by gallic acid. MCF-7 cells transfected with control siRNA or siRNA for KDM2A were treated with 50 μM gallic acid for 4 hours. The levels of H3K36me2, H3K36me3, and KDM2A in the rDNA promoter were analyzed by ChIP assays. The results are expressed as fold changes of the values with various conditions to those in cells cultured with neither gallic acid nor DMS. All experiments were performed three times (n=3), and the mean values with standard deviations are indicated. *; $P<0.05$.

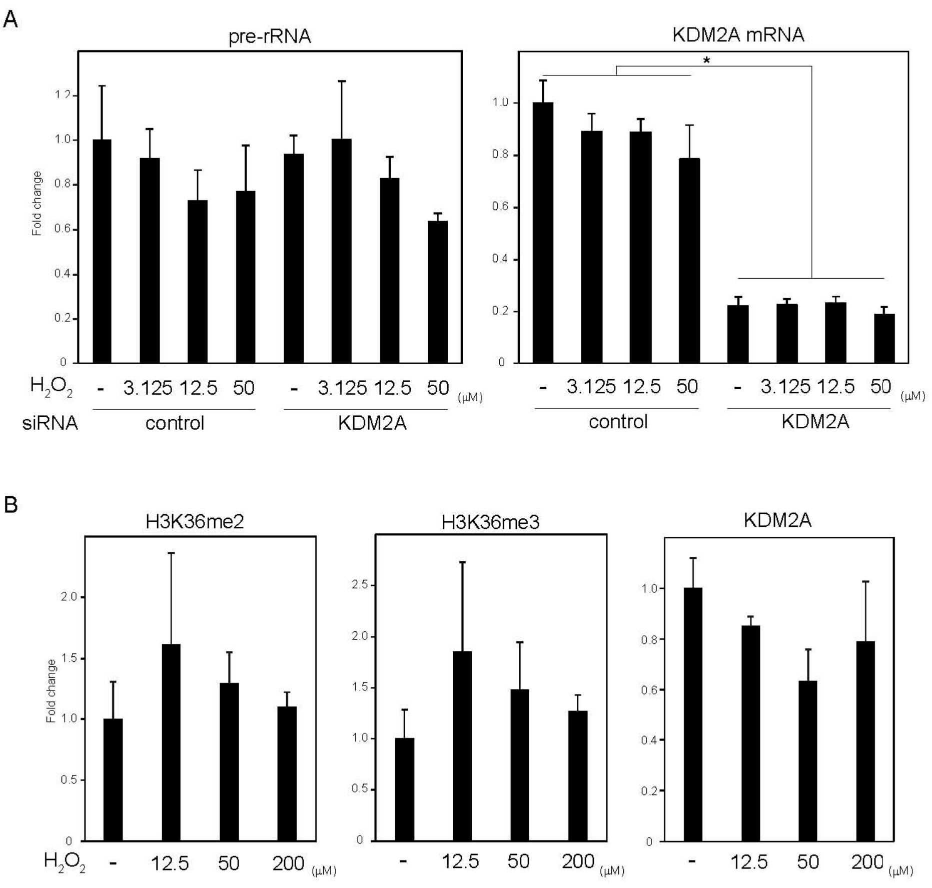

Figure S3 Tanaka et al.

Figure S3. H_2O_2 does not activate the demethylase activity of KDM2A. A, H_2O_2 reduces rRNA transcription slightly depending on KDM2A. MCF-7 cells transfected with siRNA for KDM2A or control siRNA were treated with or without H_2O_2 at the indicated concentrations for 4 hours. Total RNAs were isolated and analyzed by qRT-PCR to detect rRNA transcription (pre-rRNA) (left panel) and KDM2A mRNA (right panel). The ratios of the values for cells treated with various concentrations of H_2O_2 to those for cells treated with control siRNA without H_2O_2 are shown. B, H_2O_2 did not decrease the H3K36me2 mark in the rDNA promoter. MCF-7 cells were treated with or without H_2O_2 for 4 hours. The levels of H3K36me2, H3K36me3, and KDM2A in the rDNA promoter were analyzed by ChIP assay. The results are expressed as fold changes of the values with various H_2O_2 conditions to those of cells cultured without H_2O_2. All experiments were performed three times (n=3), and the mean values with standard deviations are indicated. *; $P<0.05$.

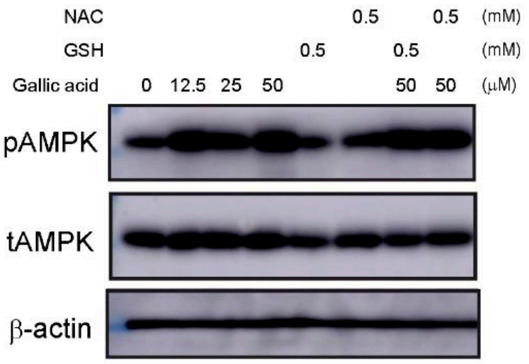

Figure S4 Tanaka et al.

Figure S4. AMPK activation by 50 μM gallic acid occurs in the presence of NAC and GSH. MCF-7 cells were treated with gallic acid in the presence or absence of NAC or GSH as indicated concentrations for 4 hours. Cells were lysed and analyzed by immunoblotting with antibodies for phosphorylated AMPKα (Thr-172), total AMPKα, and β-actin.

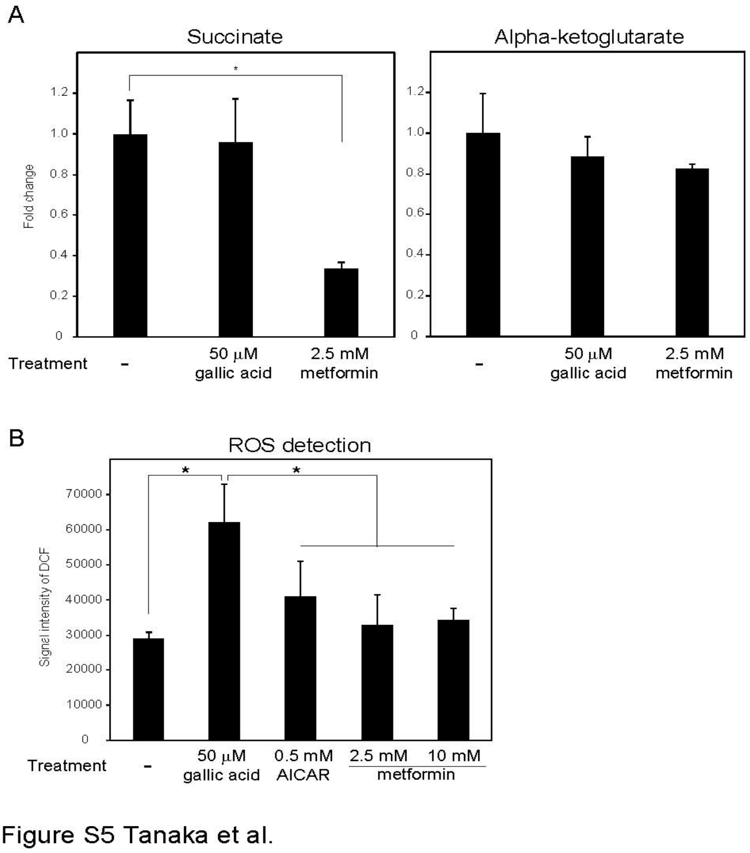

Figure S5. Levels of succinate, α-KG, and ROS in cells treated with gallic acid or metformin. A, The levels of succinate and α-KG in cells treated with 50 μM gallic acid. MCF-7 cells were treated with or without 50 μM gallic acid or 2.5 mM metformin for 4 hours. Cells were washed with PBS, and then intracellular metabolites were extracted with methanol. The levels of succinate and α-KG in the extracts were measured by mass spectrometry as previously described [9]. These levels normalized with total ion counts are shown as fold changes. B, The elevation of ROS production by gallic acid but not by other KDM2A activators. MCF-7 cells were treated with a DCFDA probe for 45 min. After cells were washed with PBS, cells were treated with 50 μM gallic acid, 0.5 mM AICAR, 2.5 mM metformin, or 10 mM metformin for 4 hours. The signal intensities for DCF probe were measured by a plate reader with a green filter set. The signals after subtraction of background values are shown. All experiments were performed more than three times, and the mean values with standard deviations are indicated. *; $P<0.05$.

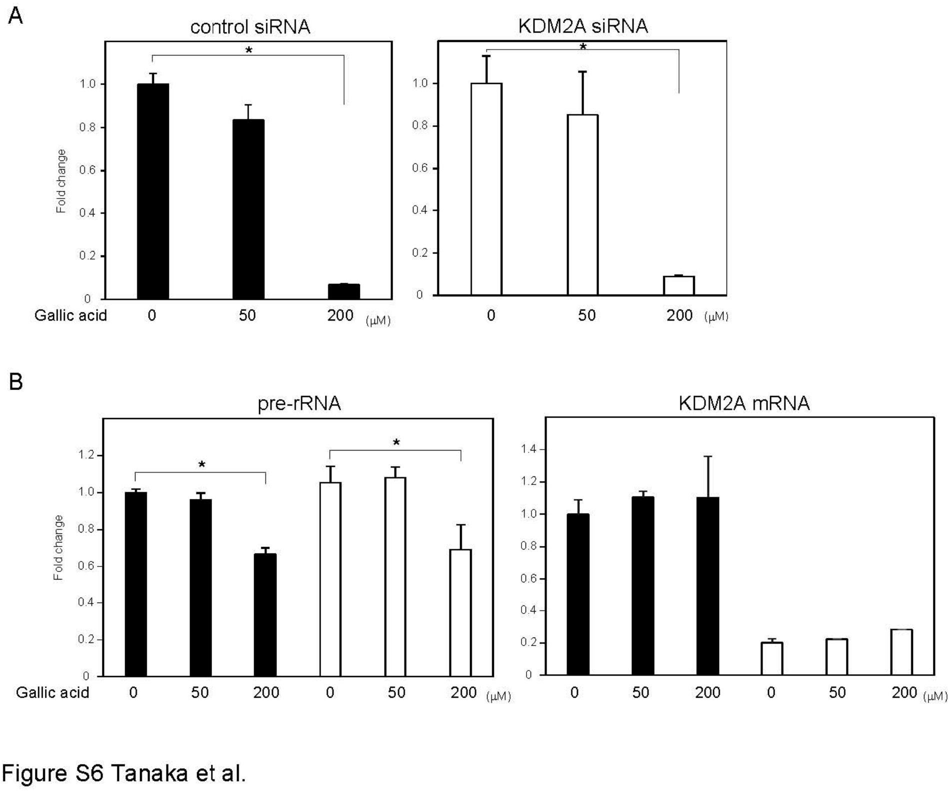

Figure S6. Gallic acid does not reduce rRNA transcription through KDM2A in MCF10A cells. A, Reduction of cell proliferation by gallic acid in MCF10A cells. MCF10A cells transfected with control siRNA (left panel) or siRNA for KDM2A (right panel) were treated with 0, 50, or 200 μM gallic acid. After culture for 2 days, cell numbers were evaluated by a CyQUANT Direct Cell Proliferation Assay to measure the DNA amounts. The results are shown as fold changes of the values with compounds to that without gallic acid. B, Reduction of rRNA transcription by gallic acid in MCF10A cells. MCF10A cells transfected with control siRNA (white bars) or siRNA for KDM2A (black bars) were treated with 0, 50, and 200 μM gallic acid for 4 hours. Total RNAs were isolated and analyzed by qRT-PCR to detect rRNA transcription (pre-rRNA) (left panel) and KDM2A mRNA (right panel). The ratios of the values for cells treated with gallic acid to those for cells treated without gallic acid are shown. All experiments were performed more than three times (n=3), and the mean values with standard deviations are indicated. *; $P<0.05$.

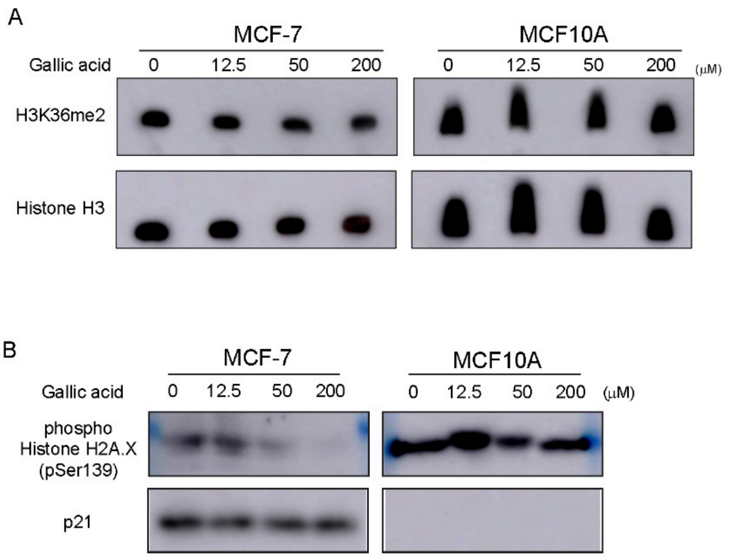

Figure S7 Tanaka et al.

Figure S7. The effects of gallic acid on the levels of histone H3K36me2, p21, and γH2A.X. A, The effects of gallic acid on the levels of H3K36me2 in MCF-7 and MCF10A cells. MCF-7 and MCF10A cells were treated with gallic acid at indicated concentrations for 4 hour, then cells were lysed and applied to western blot analysis to detect H3K36me2 and histone H3 using anti-H3K36me2 specific antibody (MAB Institute, Inc.; #MABI0332-100) and anti-H3 specific antibody (Abcam; # ab1791), respectively. The levels of H3K36me2 and histone H3 were hardly changed by gallic acid treatment in MCF-7 and MCF10A cells. B, The effects of gallic acid on the levels of γH2A.X and p21 to monitor double strand breaks (DBSs) response in MCF-7 and MCF10A cells. MCF-7 and MCF10A cells were treated with gallic acid at indicated concentrations for 4 hours, then cells were lysed and applied to western blot analysis to detect p21 and phosphorylated histone H2A.X (γH2A.X) using anti-p21 specific antibody (Abcam; #ab109119) and anti-γH2A.X specific antibody (γH2A.X, Ser 139, Upstate; #16-193), respectively. The levels of p21 were not increased by gallic acid treatment in MCF-7 cells. p21 was not detected in MCF10A cells in the conditions here. The levels of γH2A.X were decreased in MCF-7 cells, depending on the increased gallic acid concentrations. The levels of γH2A.X in MCF10A cells were hardly affected by gallic acid treatment.

© 2020 by the authors. Licensee MDPI, Basel, Switzerland. This article is an open access article distributed under the terms and conditions of the Creative Commons Attribution (CC BY) license (http://creativecommons.org/licenses/by/4.0/).
